# Supplementary material for: Integrating national surveys to estimate small area variations in poor health and limiting long-term illness in Great Britain
Source: BMJ Open. 2017 Aug 28;7(8):e016936. doi: 10.1136/bmjopen-2017-016936 (PMC5724299; doi:10.1136/bmjopen-2017-016936)
Supplement: Author's manuscript [file bmjopen-2017-016936.draft_revisions.pdf]

BMJ Open

Integrating national surveys to estimate small area variations in poor health and limiting long term illness

Journal:	*BMJ Open*
Manuscript ID	bmjopen-2017-016936
Article Type:	Research
Date Submitted by the Author:	27-Mar-2017
Complete List of Authors:	Moon, Graham; University of Southampton, Geography and Environment<br>Aitken, Grant; ISD Scotland<br>Taylor, Joanna; Independent Scholar<br>Twigg, Liz; University of Portsmouth
**Primary Subject Heading**:	Research methods
Secondary Subject Heading:	Public health
Keywords:	Small Area Estimation, Survey Methodology, Self-rated General Health, Limiting Long-term Illness

SCHOLARONE™ Manuscripts

For peer review only

Integrating national surveys to estimate small area variations in poor health and limiting long term illness

Graham Moon (*)
Geography and Environment, University of Southampton, Highfield, Southampton SO17 1BJ, UK
(g.moon@soton.ac.uk; tel. 02392 843822)

Grant Aitken
Information Services Division, NHS Scotland, UK

Joanna Taylor
Independent Scholar, Petersfield, UK

Liz Twigg
Department of Geography, University of Portsmouth, UK

(*) Corresponding Author

Keywords

Small Area Estimation; Survey Methodology; Self-rated General Health ; Limiting Long-term Illness

Word count: 3405 excluding title page, references, figures and tables

For peer review only

Integrating national surveys to estimate small area variations in poor health and limiting long term illness

Abstract

Objectives: This study aims to address, for the first time, the challenges of constructing small area estimates of health status using linked national surveys. The study also seeks to assess the concordance of these small area estimates with data from national censuses.

Setting: Population level health status in England, Scotland and Wales

Participants: A linked integrated dataset of 23,374 survey respondents (16+ years) from the 2011 waves of the Health Survey for England (n=8,603), the Scottish Health Survey (n=7,537) and the Welsh Health Survey (n=7,234)

Primary and secondary outcome measures: Population prevalence of poorer self-rated health and limiting long term illness. A multilevel small area estimation modelling approach was used to estimate prevalence of these outcomes for Middle Super Output Areas in England and Wales and Intermediate Zones in Scotland. The estimates were then compared to matched measures from the contemporaneous 2011 UK Census.

Results: There was a strong positive association between the small area estimates and matched Census measures for all three countries for both poorer self-rated health (r=0.828, 95% CI 0.821 - 0.834) and limiting long-term illness (r=0.831, 95% CI 0.824 to 0.837), although systematic differences were evident and small area estimation tended to indicate higher prevalences than Census data.

Conclusions: Despite strong concordance, variations in the small area prevalences of poorer self-rated health and limiting long-term illness evident in Census data cannot be replicated perfectly using small area estimation with linked national surveys. This reflects a lack of harmonisation between surveys over question wording and design. The nature of small area estimates as 'expected values' also needs to be better understood.

Article Summary

Strengths and limitations of this study

- Reports on small area estimates of health status derived from the novel linkage of three large well-designed routine national health surveys
- Develops and enhances a well-reputed approach to the small area estimation of health indicators
- Highlights the enduring challenges entailed in harmonising measures of health status across different surveys
- Enhances understanding of the reasons that underlie discrepancies between small area estimates and equivalent observed data

For peer review only

Introduction

Small area data on variations in health status have long been seen as central to geographical comparisons of health needs [1]. Effectiveness is enhanced when data are available at very local scales facilitating sensitive, community-focussed action. Such data additionally need to be provided to consistent, robust and reliable standards that are shared nationally and, ideally, internationally. Moreover they need to capture the health status of whole populations, to ensure comprehensive rather than selective coverage. These ideals are seldom met. National surveys are not usually designed to provide valid data down to suitably small areas, although an exception is offered in the few cases where national censuses provide small area health data. Local surveys, if they exist at all, can vary substantially in design, making comparisons difficult. Using administrative data to make local estimates restricts attention to users of services and thus lacks population representativeness.

To address the unmet need for small area data, researchers across the world have increasingly turned to small area estimation (SAE) methods [2]. SAE methods use statistical or mathematical methods to manipulate national survey data to produce local estimates for a target measure. A common methodological root is the association in a national survey between a target variable of interest and covariates thought to predict that target variable. Estimation uses local data on the covariates. The SAE process generally takes place within a single national setting with multiple surveys being used to enhance the pool of available covariates. There has been limited attention to linking surveys from different geographical contexts to enhance the spatial coverage of estimates through a larger pool of cases. Addressing this omission recognises that health issues seldom respect geographical borders. It also enables the identification of ‘place effects’ on outcome measures and reveals the extent to which definitions of key health variables differ with geographical setting.

Health applications of SAE have focussed mainly on outcomes and behaviours [3-7]. Rather less attention has been given to SAEs of general measures of health status such as Limiting Long-Term Illness (LLTI) and Self-reported General Health (SRGH). Such measures are good at picking up ageing

For peer review only - http://bmjopen.bmj.com/site/about/guidelines.xhtml

populations and chronic illness as well as pockets of severe health deprivation [8-13]. For these reasons, they are frequently included in national censuses. Though this might be held to obviate the need for SAEs of health status, it presents an opportunity to address a frequent criticism of SAEs: the absence of validation. Small area census data on health status provide what is arguably a 'gold standard' against which SAEs can be compared [14].

Motivated by the lack of previous research on health-related SAE using surveys from more than one geographical setting and the opportunity to ground-truth SAEs of health status using small area census data, this paper addresses two objectives. First, we develop parsimonious SAE models of LLTI and SRGH using surveys from multiple geographical settings. Second, the estimates from these models are compared to population census data. As both SAEs and census data are population-level measures, we hypothesise a close concordance.

Methods

We created SAEs by modelling routine data and applying the coefficients from our models to census data on the covariate measures. Our SAEs were then compared to census data on LLTI and SRGH. Great Britain was the setting for the study as we had ready access to the separate surveys for the constituent countries of Wales, Scotland and England as well as to census data for each country. LLTI and SRGH are established measures of health status in each country. The UK Census began collecting information on SRGH and LLTI in 1991.

Small Area Estimation

SAE models were derived primarily from a linked data file comprising the individual responses to the 2011 versions of the Health Survey for England (HfSE), the Scottish Health Survey (SHS) and the

For peer review only - http://bmjopen.bmj.com/site/about/guidelines.xhtml

Welsh Health Survey (WHS). These sources were accessed through the UK Data Service. Surveys from 2011 were selected to facilitate direct comparison with 2011 decennial population census. Full details of the conduct of the surveys, the measurement of variables and response rates are given in the 2011 HSfE, SHS and WHS reports [15-17]. Collectively the sources offer comprehensive well-found authoritative insights into health status in each country. After data cleaning to address missing data the working data file comprised 23,374 individuals (aged 16+ years; England n=8,603, Scotland n=7,537, Wales n=7,234).

Data from the three health surveys were supplemented with linked data on disability and multiple deprivation at the area level, in recognition of the close association between these factors and our target variable [8, 10, 18]. We obtained special permission to use geo-coded data to link these additional variables for Middle (layer) Super Output Areas (MSOAs). MSOAs (known as Intermediate Zones in Scotland, but referred to as MSOAs throughout this paper) total 6,791 in England, 1,235 in Scotland and 410 in Wales. We added the combined rate of Disability Living Allowance and Attendance Allowance per 1,000 adults per MSOA, and the combined rate of Incapacity Benefit plus Severe Disablement Allowance plus Employment and Support Allowance per 1,000 adults per MSOA. Both benefit measures were derived from the Department of Work and Pensions (DWP) website [19]. For deprivation we used a UK-wide Index of Multiple Deprivation (IMD) score, calibrated from the English, Scottish and Welsh versions of the index using a modified version of the method outlined by Payne and Abel. [20]. To track survey effects we also added a flag denoting whether a respondent was from England, Scotland or Wales.

The outcome measures for our two SAE models were poorer SRGH and possession of an LLTI. Both measures required harmonisation of the relevant questions across the three surveys and in the Decennial Population Census (Table 1). For SRGH, respondents were asked to self-report their general health. In the Census, HSfE and SHS 'very good' or 'good' general health was coded as good SRGH; 'fair', 'bad' or 'very bad' general health was coded as poorer SRGH. In the WHS 'excellent',

For peer review only - http://bmjopen.bmj.com/site/about/guidelines.xhtml

For peer review only

'very good' or 'good' general health was coded as good SRGH and 'fair' or 'poor' general health was coded as poorer SRGH. LLTI was also dichotomised. In the Census and the WHS, respondents were asked first to define if they had any long-term illness, health problem or disability which limited their daily activities or the work they could do. If they responded 'yes, limited a lot' or 'yes, limited a little' they were coded as having a LLTI, and if they responded 'no' they were not. In the HSfE people were asked if they had a long-standing illness, disability or infirmity troubling them over a period of time. If they responded yes, they were then asked if this limited their activities in any way, with those individuals answering yes to this second question being coded as having a LLTI. In the SHS people were if they had a long-standing physical or mental condition or disability that troubled them for at least 12 months. If they responded yes, they were asked if this condition limited their activities in any way, with those individuals answering yes to this question coded as having a LLTI.

Table 1: Wording of SRGH and LLTI questions for Census, HSfE, SHS and WHS for people aged 16+

	**SRGH: question and responses**			
**2011 Census**	How is your health in general?	Very Good or Good	Fair	Bad or Very Bad
**2011 Health Survey for England**	How is your health in general? Would you say it was….	Very Good or Good	Fair	Bad or Very Bad
**2011 Scottish Health Survey**	How is your health in general? Would you say it was…..	Very Good or Good	Fair	Bad or Very Bad
**2011 Welsh Health Survey**	In general, would you say your health is?	Excellent, Very Good or Good	Fair	Poor
	**LLTI: question and responses**			
**2011 Census**	Are your day-to-day activities limited because of a health problem or disability which has lasted, or is expected to last, at least 12 months? Include problems related to old age.	No	Yes, limited a little	Yes, limited a lot
**2011 Health Survey for England**	Do you have any long-standing illness, disability or infirmity? By long-standing I mean anything that has troubled you over a period of time, or that is likely to affect you over a period of time?	No	Yes	
	If yes, does this illness or disability/do any of these illnesses or disabilities limit your activities in any way?	No	Yes	
**2011 Scottish Health Survey**	Do you have a long-standing physical or mental condition or disability that has troubled you for at least 12 months, or that is likely to affect you for at least 12 months?	No	Yes	
	If yes, does (name of condition) limit your activities in any way?	No	Yes	
**2011 Welsh Health Survey**	Are your day-to-day activities limited because of a health problem or disability which has lasted, or is expected to last, at least 12 months? Include problems related to old age.	No	Yes, limited a little	Yes, limited a lot

For peer review only - http://bmjopen.bmj.com/site/about/guidelines.xhtml

Covariate data to facilitate the generation of small area estimates was derived from the linked data file. A parsimonious selection of covariates was made reflecting the known associations between LLTI and SRGH and age, sex, disability and deprivation [21, 22]. Individual-level age and sex were self-reported measures. Age was grouped into thirteen categories: those aged 16 to 19; 5 year age groups until the age of 74; and all individuals aged 75+. These individual-level covariates were supplemented with the area-level measures of disability and deprivation introduced above.

To create SAEs we used the established multilevel small area estimation process recognising the hierarchical structure of the source surveys [3, 23, 24]. Initial data management into individual and area level covariates used IBM SPSS Statistics (V22). We then developed 2-level logistic models of individuals nested within MSOAs using MLwiN (V2.35) [25, 26]. Separate models were produced to estimate SRGH and LLTI. Both models initially used iterative generalised least squares with first order maximum quasi-likelihood estimation. Once a final model was selected, a Monte Carlo Markov Chain (MCMC) approach was used to refine the model and allow for more robust estimates and standard errors. Both MCMC models were run through 500,000 iterations, with an initial burn-in period of 50,000 iterations applied. Models were tested for interactions between the age and sex terms. Area-level variables were centred on their grand mean. Variables were retained in the final models if they were found to be statistically significant using the Chi-Squared test. SAEs of SRGH and LLTI at MSOA level were produced by applying the model beta coefficients to a data file for all MSOAs in Great Britain comprising cross-tabulations of age and sex from the 2011 Decennial Population Census together with the area-level indicators. We calculated 95% credible intervals for each estimate.

Census Comparison

In the UK, the 2011 Census is the most recent source of information on local inequalities in SRGH and LLTI. We compared our MSOA SAEs to census data on SRGH and LLTI for MSOAs. For England

For peer review only - http://bmjopen.bmj.com/site/about/guidelines.xhtml

and Wales this was sourced from the ONS official labour market statistics website. For Scotland it was downloaded from the Scottish Census data warehouse.

SAEs and Census estimates were compared using regression and correlation analysis following the Scarborough methodology used previously for validating SAEs for coronary heart disease [27]. SAEs were plotted against the Census measurement of the same target variable at the MSOA level. Convergent validity was achieved if the line of best fit had a gradient with confidence intervals including one and an intercept with confidence intervals including zero. For each model, we considered four regression lines: one for Great Britain as a whole and one each for England, Scotland and Wales. We also sought high correlations.

Results

A descriptive summary of the full linked data file used to construct the SAEs is set out in Table 2. The resulting small area estimation models are shown in Table 3. We show logits and standard errors as these provide the input to the SAE process. Having poor SRGH was largely a function of increasing age, and higher MSOA IMD scores (increased deprivation). A similar picture was evident for LLTI with being female playing an additional role. MSOA-level benefit measures had little effect on either SRGH or LLTI. For SRGH the models showed no difference between Scotland and the reference country of England but a markedly lower likelihood of poorer SRGH in Wales. In contrast, being located in either Wales or Scotland was associated with a higher likelihood of reporting an LLTI compared to England.

For peer review only - http://bmjopen.bmj.com/site/about/guidelines.xhtml

Table 2: Descriptive statistics: full linked data set

Variable	Category	Full Sample		General Health		LLTI sample	
		Number	Column %	Number	Column %	Number	Column %
All	Aged 16+	23 374	100	23 355	100	23 281	100
Country	England	8 603	36.8	8 603	36.8	8 603	37.0
	Scotland	7 537	32.3	7 537	32.3	7 537	32.4
	Wales	7 234	30.9	7 215	30.9	7 141	30.5
Sex	Male	10 285	44.0	10 276	44.0	10 240	44.0
	Female	13 089	56.0	13 079	56.0	13 041	56.0
Age	16-19	772	3.3	770	3.3	780	3.4
	20-24	1 146	4.9	1 146	4.9	1 153	5.0
	25-29	1 403	6.0	1 403	6.0	1 404	6.0
	30-34	1 650	7.1	1 650	7.1	1 645	7.1
	35-39	1 790	7.7	1 788	7.7	1 783	7.7
	40-44	2 123	9.1	2 122	9.1	2 112	9.1
	45-49	2 140	9.2	2 138	9.2	2 136	9.2
	50-54	2 032	8.7	2 030	8.7	2 023	8.7
	55-59	1 941	8.3	1 940	8.3	1 935	8.3
	60-64	2 272	9.7	2 271	9.7	2 268	9.7
	65-69	1 917	8.2	1 914	8.2	1 907	8.2
	70-74	1 509	6.5	1 508	6.5	1 494	6.4
	75+	2 679	11.4	2 675	11.4	2 641	11.3
General Health	Fair/poor/ very poor	-	-	5 956	25.5	-	-
	Very good/ good	-	-	17 399	74.5	-	-
LLTI	Present	-	-	-	-	7 309	31.4
	Not present	-	-	-	-	15 972	68.6
		Mean (SD)		Mean (SD)		Mean (SD)	
Disability Benefits		109.39 (±45.19)		109.38 (±45.20)		109.18 (±45.13)	
Work Benefits		86.66 (±57.49)		86.68 (±57.51)		86.73 (±57.60)	
IMD		19.11 (±10.67)		19.10 (±10.67)		19.07 (±10.67)	

For peer review only - http://bmjopen.bmj.com/site/about/guidelines.xhtml

Table 3: SRGH and LLTI model variables, logit and standard error values

	Poorer SRGH model		LLTI model	
	Logit	Standard Error	Logit	Standard Error
**Constant**	-2.470	0.137	-2.422	0.122
**Sex: Female**	0.059	0.032	0.151	0.031
**Age: 20-24**	0.188	0.165	-0.193	0.156
**Age: 25.29**	0.339	0.157	0.178	0.143
**Age: 30-34**	0.208	0.156	0.173	0.140
**Age: 35-39**	0.680	0.148	0.455	0.135
**Age: 40-44**	0.863	0.144	0.654	0.131
**Age: 45-49**	1.168	0.142	0.984	0.129
**Age: 50-54**	1.465	0.142	1.281	0.128
**Age: 55-59**	1.650	0.142	1.436	0.128
**Age: 60-64**	1.758	0.140	1.671	0.127
**Age: 65-69**	1.886	0.141	1.816	0.128
**Age: 70-74**	2.086	0.143	2.023	0.130
**Age: 75+**	2.541	0.138	2.617	0.126
**IMD**	0.023	0.003	0.011	0.003
**Disability Benefits**	0.002	0.001	0.002	0.001
**Work Benefits**	-0.001	0.001	-	-
**Scotland**	0.150	0.110	0.319	0.046
**Wales**	-0.456	0.056	0.469	0.049

Summary statistics on the SAEs derived from these models are presented for England, Scotland and Wales in Table 4. The mean MSOA prevalence of poorer SRGH is highest in Scotland and lowest in Wales. For LLTI, Wales has the highest mean MSOA prevalence and England is lowest. In both Scotland and Wales, the mean MSOA prevalence for LLTI is notably higher than that for poorer SRGH. Table 4 also indicates the mean MSOA census prevalences for poorer SRGH and LLTI for each country, providing initial insights into the match between SAEs and 'gold standard' census data. Differences are evident. For poorer SRGH, Scotland has the lowest mean prevalence on the Census measure but the highest on SAE measure; for Wales the situation is reversed. Both England and Scotland have a lower mean prevalence on the Census measure compared to the SAE while for Wales the SAE prevalence is higher. In the case of LLTI the relative position of the three countries is the same for the two measures but the Census mean prevalences are substantially lower.

For peer review only - http://bmjopen.bmj.com/site/about/guidelines.xhtml

Table 4: MSOA level SAEs and Census estimates compared

	Poorer SRGH					LLTI				
	Census mean	SAE				Census mean	SAE			
		Mean	SD	Min	Max		Mean	SD	Min	Max
**England**	22.04	25.92	5.53	10.64	55.76	21.48	25.23	4.09	9.4	43.96
**Scotland**	21.33	29.17	6.59	11.89	54.62	23.10	31.47	4.86	14.31	48.33
**Wales**	26.23	21.65	5.23	6.68	41.36	27.76	37.29	4.97	13.97	51.54

All figures are percentages except SD (standard deviation)

The concordance between SAEs and Census data is explored further in Figure 1. The diagonal reference line captures the scenario where the SAE and Census estimates would match. The SAEs for poorer SRGH in England and Scotland were higher for most small areas compared to the Census, while the great majority of Welsh estimates were lower. For LLTI most SAE prevalences exceeded the equivalent Census prevalences; those MSOAs with SAEs less than the corresponding Census values tended to be in England.

For peer review only

Figure 1: SAEs versus Census Estimates: LLTI (top), poorer SRGH (bottom)

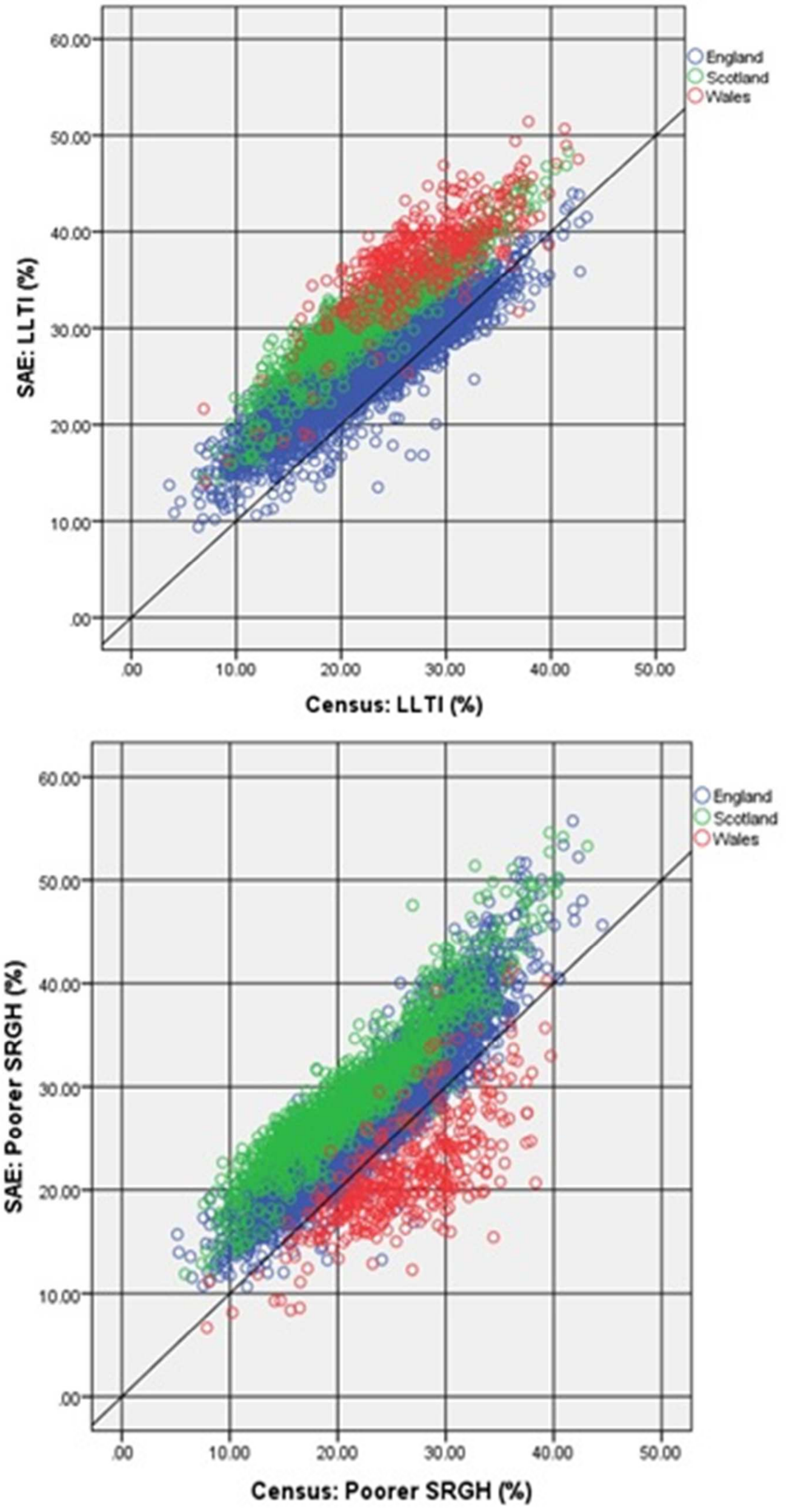

For peer review only - http://bmjopen.bmj.com/site/about/guidelines.xhtml

Table 5 continues the exploration of the match between SAEs and Census using the 'Scarborough criteria'. There are strong correlations between the two measures across all settings suggesting broad agreement. However, neither for individual countries nor collectively do any of the SAEs exhibit anything approaching the requirements for a strong concordance with Census measures. All have intercepts significantly above zero and gradients that depart markedly from one.

Table 5: Convergent validity of SAEs against Census estimates at MSOA level

	Intercept	Lower CI	Upper CI	Contain Zero?	Gradient	Lower CI	Upper CI	Contain One?	Correlation
**Poorer SRGH**									
**GB**	6.87	6.58	7.16	X	0.87	0.86	0.89	X	0.828**
**England**	4.98	4.75	5.21	X	0.95	0.94	0.96	X	0.916**
**Scotland**	8.64	8.18	9.10	X	0.96	0.94	0.98	X	0.933**
**Wales**	3.76	2.04	5.49	X	0.68	0.62	0.75	X	0.719*
**LLTI**									
**GB**	9.24	8.99	9.50	X	0.79	0.78	0.81	X	0.832**
**England**	10.56	10.36	10.74	X	0.68	0.67	0.69	X	0.895**
**Scotland**	13.31	12.87	13.74	X	0.79	0.77	0.81	X	0.922**
**Wales**	17.98	16.48	19.47	X	0.69	0.64	0.75	X	0.789*

*significant p<0.05
**significant p<0.01

Discussion

Even with jurisdictions as closely associated as England, Scotland and Wales and ongoing UK-wide attempts at harmonising questions in health and other routine national surveys, there remain differences in wording and format that pose difficulties for SAE when it comes to linking survey input data across geographical settings. Overcoming these difficulties requires compromise and accommodation but is possible. In response to our first objective, we have shown how a cross-setting dataset can be developed as the basis for a parsimonious SAE model reflecting the key social determinants of poorer SRGH and LLTI.

Our second objective entailed comparing our SAEs with matched and contemporaneous MSOA Census data. Here our results were at best equivocal. While strong correlations were evident,

For peer review only - http://bmjopen.bmj.com/site/about/guidelines.xhtml

suggesting a tight association between the two measures running in an expected direction, closer inspection revealed that SAEs tended broadly to exceed Census estimates, and MSOAs in different national contexts returned starkly varying results across the two measures. There was no evidence for a close concordance between the two measures.

Despite this lack of concordance, our findings update previous knowledge on SRGH and LLTI in Great Britain. Levels of LLTI were found to be higher in Wales than in Scotland or England using 1991 Census data [28]. This situation still persists and is evident in our SAEs, which run counter to suggestions that models under-predict LLTI in Wales but possibly confirm a continued tendency to over-prediction in Scotland [29]. 2001 Census data was used to highlight higher rates of poor SRGH in Wales compared to the rest of Great Britain [30]. Another study with 2001 Census data confirmed that levels of poorer SRGH were worst in Wales, followed by Scotland, then England [31]. Our analysis sustains this finding with 2011 Census data but our SAEs suggest a contrary picture in which Wales has better levels of poorer SRGH than either Scotland or England.

Reasons for the differences between our SAEs and contemporaneous Census data for matched measures are of relevance to future research on SAE and merit examination. Differences in the wording of the SRGH and LLTI questions were evident between the source surveys and between the surveys and the Census. There were also variations in the categorisation of outcome possibilities and in the positioning of the questions within the surveys. In the English and Scottish surveys the SRGH question appeared at the start of the individual interview immediately after age, with the LLTI question appearing immediately afterwards . In the WHS, additional health questions were asked before the SRGH questions . Asking about SHRG after, rather than before, other health questions, results in more positive health assessments and replacing ‘very good’ with ‘excellent’ as a response category has a similarly more positive effect [32]. The wording of the WHS question about LLTI and specifically the inclusion of the reference to ‘problems of old age’ may have led to greater levels of problem identification [9] [11]. Collectively there points echo calls from other jurisdictions for

For peer review only - http://bmjopen.bmj.com/site/about/guidelines.xhtml

continued work on the harmonisation of questions between surveys and censuses across national borders [33].

Another reason for discrepancies between our SAEs and Census data is that Census and survey information are collected in different ways. The householder is responsible for ensuring that the UK Census is completed whereas the surveys are completed by an individual. Furthermore the UK Census is a self-completion form but our surveys were interviewer administered. Both these factors can lead to Census prevalence data generally being lower that that from surveys [34]. One manifestation of both this and the previous point is that the input survey data for our SAE models (Table 2) collectively evidenced prevalences of 25.5% for poorer SRGH and 31.4% for LLTI, both greater than all bar one of the corresponding national prevalences evident for Census data in Table 3. Inevitably the SAEs accord more with this input data.

Though soundly based on theory, our models were undeniably simple. Research using a multinomial LLTI outcome measures has found improved concordance with 2011 Census estimates at MSOA level in England [35]. Additional socio-economic, health and clinical covariates, known to be associated with increased SRGH or LLTI prevalence were not included within the modelling framework due to data constraints and the requirements of the SAE process. These omissions may have been significant and uncaptured by the areal IMD and benefit measures. For example there may be systematic differences between social groups in their understanding of SRGH and the omission of ethnicity is undoubtedly significant [36].

As a final point, we should reflect on the nature of SAEs and Census data. The former are perhaps more usefully seen as expected values for the target variable, given the covariates included in the modelling process [37, 38]. The latter are also estimates, subject to differential response and variations in understanding [39, 40]. The Census is thus not a ‘gold standard’ though it comes close. Nor should we anticipate that SAEs ought to match their equivalent census measures. Rather, they

For peer review only - http://bmjopen.bmj.com/site/about/guidelines.xhtml

provide an expectation against which Census data can be compared. In our analysis, linking surveys across three countries points to an expectation that levels of poorer SRGH and LLTI would, given variations in population distributions by age and sex, and the varying areal prevalences of benefit take-up and deprivation, be rather higher than suggested by Census data.

Funding Statement

This work was supported by the UK Economic and Social Research Council grant number ES/K003046/1.

Author Contributions

GM and LT conceived and led the study, and participated in data analysis and interpretation. JT led on data acquisition and participated in data analysis. GA participated in data analysis and interpretation. All authors contributed to drafting, revision and final approval of the manuscript. All authors are responsible for the manuscript.

Competing Interests

There were no competing interests

Data Sharing

Project data files are available by emailing the corresponding author

For peer review only

For peer review only - http://bmjopen.bmj.com/site/about/guidelines.xhtml

References

1 Mooney A, Rives N. Measures of community health status for health planning. *Health Services Research* 1978;**13**:129-45.
2 Rao J, Molina I. *Small Area Estimation*. London: John Wiley & Sons 2015.
3 Twigg L, Moon G. Predicting small area health-related behaviour: a comparison of multilevel synthetic estimation and local survey data. *Social Science and Medicine* 2002;**54**:931-7.
4 Dwyer-Lindgren L, Mokdad AH, Srebotnjak T*, et al.* Cigarette smoking prevalence in US counties: 1996-2012. *Population Health Metrics* 2014;**12**.
5 Ha NS, Lahiri P, Parsons V. Methods and results for small area estimation using smoking data from the 2008 National Health Interview Survey. *Statistics in Medicine* 2014;**33**:3932-45.
6 Wang Y, Ponce NA, Wang P*, et al.* Generating health estimates by zip code: A semiparametric small area estimation approach using the California health interview survey. *American Journal of Public Health* 2015;**105**:2534-40.
7 Zhang X, Holt JB, Yun S*, et al.* Validation of Multilevel Regression and Poststratification Methodology for Small Area Estimation of Health Indicators from the Behavioral Risk Factor Surveillance System. *American Journal of Epidemiology* 2015;**182**:127-37.
8 Marshall A. Developing a methodology for the local estimation and projection of limiting long term illness and disability. *PhD Thesis*: University of Manchester 2009.
9 Cohen G, Forbes J, Garraway M. Interpreting self reported limiting long term illness. *BMJ* 1995;**311**:722.
10 Martin S, Sheldon TA, Smith P. Interpreting the new illness question in the UK census for health research on small areas. *Journal of Epidemiology and Community Health* 1995;**49**:634-41.
11 Jordan K, Ong BN, Croft P. Researching limiting long-term illness. *Social Science and Medicine* 2000;**50**:397-405.
12 Jordan K, Ong BN, Croft P. Previous consultation and self reported health status as predictors of future demand for primary care. *Journal of Epidemiology and Community Health* 2003;**57**:109-13.
13 Taylor J, Twigg L, Moon G. The convergent validity of three surveys as alternative sources of health information to the 2011 UK census. *Social Science and Medicine* 2014;**116**:187-92.
14 Taylor J, Moon G, Twigg L. Using geocoded survey data to improve the accuracy of multilevel small area synthetic estimates. *Social Science Research* 2016;**56**:108-16.
15 NatCen Social Research. Health Survey for England 2011 user guide. London: NatCen 2012.
16 Rutherford L, Sharp C, Bromley C, eds. *The Scottish Health Survey 2011 edition volume 3 technical report*. Edinburgh: A National Statistics Publication for Scotland 2012.
17 NatCen Social Research. Welsh Health Survey 2012 - Technical Report London: NatCen 2012a.
18 Payne N, Saul C. What common disorders do those reporting limiting long-term illness experience, and what is their survival and health service utilization experience? *Journal of Public Health Medicine* 2000;**22**:324-9.
19 DWP. Welcome to Neighbourhood Statistics Data. 2013.
20 Payne R, Abel G. UK indices of multiple deprivation - a way to make comparisons across constituent countries easier. London: Office for National Statistics 2012.
21 Martin D, Wright JA. Disease prevalence in the English population: A comparison of primary care registers and prevalence models. *Social Science and Medicine* 2009;**68**:266-74.
22 Zhang X, Cook P, Lisboa P*, et al.* The effects of deprivation and relative deprivation on self-reported morbidity in England: An area-level ecological study. *International Journal of Health Geographics* 2013;**12**:5.
23 Twigg L, Moon G, Jones K. Predicting small-area health-related behaviour: a comparison of smoking and drinking indicators. *Social Science & Medicine* 2000;**50**:1109-20.

For peer review only - http://bmjopen.bmj.com/site/about/guidelines.xhtml

24 Moon G, Quarendon G, Barnard S*, et al.* Fat nation: Deciphering the distinctive geographies of obesity in England. *Social Science and Medicine* 2007;**65**:20-31.

25 Rasbash J, Charlton C, Browne W*, et al.* MLwiN Version 2.35. Centre for Multilevel Modelling, University of Bristol. 2015.

26 Browne W. MCMC Estimation in MLwiN. Centre for Multilevel Modelling, University of Bristol 2009.

27 Scarborough P, Allender S, Rayner M*, et al.* Validation of model-based estimates (synthetic estimates) of the prevalence of risk factors for coronary heart disease for wards in England. *Health & Place* 2009;**15**:596-605.

28 Gould M, Jones K. Analyzing perceived limiting long-term illness using UK census microdata. *Social Science & Medicine* 1996;**42**:857-69.

29 Senior ML. Area Variations in Self-perceived Limiting Long Term Illness in Britain, 1991: Is the Welsh Experience Exceptional?, . *Regional Studies* 1998;**32**:265-80.

30 Doran T. Is there a north-south divide in social class inequalities in health in Great Britain? Cross sectional study using data from the 2001 census. *BMJ* 2004:1043.

31 O'Reilly D. Self reported health and mortality: ecological analysis based on electoral wards across the United Kingdom. *BMJ* 2005;**331**:938.

32 Bowling A, Windsor J. The effects of question order and response-choice on self-rated health status in the English Longitudinal Study of Ageing (ELSA). *J Epidemiol Community Health* 2008;**62**:81-5.

33 Desesquelles AF, Egidi V, Salvatore MA. Why do Italian people rate their health worse than French people do? An exploration of cross-country differentials of self-rated health. *Social Science & Medicine* 2009;**68**:1124-8.

34 Gnambs T, Kaspar K. Disclosure of sensitive behaviors across self-administered survey modes: A meta-analysis. *Behavior Research Methods* 2015;**47**:1237-59.

35 Moon G, Twigg L, Taylor J. Estimating local variations in the prevalence of limiting long-term illness: evaluating multinomial small area synthetic estimation. *Journal of Epidemiology and Community Health* 2014;**68**:A71-A.

36 Evandrou M, Falkingham J, Feng Z*, et al.* Ethnic inequalities in limiting health and self-reported health in later life revisited. *Journal of Epidemiology and Community Health* 2016;**70**:653-62.

37 Twigg L, Moon G, Walker S. The smoking epidemic in England. London: Health Development Agency 2004.

38 Moon G, Altankhuyag G, Barnard S*, et al.* Smoking Prevalence in Scotland: 2003/4 sub-national estimates. NHS Health Scotland, Glasgow 2006.

39 Baffour B, Valente P. An evaluation of census quality. *Statistical Journal of the IAOS* 2012;**28**:121-35.

40 Coleman D. The twilight of the census. *Population and Development Review* 2013;**38**:334-51.

For peer review only

STROBE Statement—checklist of items that should be included in reports of observational studies

All items checked and present – subject to adjustment for small area estimation studies

	Item No	Recommendation	Where evident
**Title and abstract**	1	(*a*) Indicate the study's design with a commonly used term in the title or the abstract	Title conveys focus on survey linkage and small area estimation
		(*b*) Provide in the abstract an informative and balanced summary of what was done and what was found	Abstract follows required format
**Introduction**			
Background/rationale	2	Explain the scientific background and rationale for the investigation being reported	Pages 1-2
Objectives	3	State specific objectives, including any prespecified hypotheses	Page 2
**Methods**			
Study design	4	Present key elements of study design early in the paper	Page 2 first paragraph of Methods section
Setting	5	Describe the setting, locations, and relevant dates, including periods of recruitment, exposure, follow-up, and data collection	Page 2-3 first two paragraphs of sub-section on small area estimation, page 5/6 on census comparison
Participants	6	*Cross-sectional study*—Give the eligibility criteria, and the sources and methods of selection of participants	Page 2-3 first two paragraphs of sub-section on small area estimation; Page 5/6 on census comparison
Variables	7	Clearly define all outcomes, exposures, predictors, potential confounders, and effect modifiers. Give diagnostic criteria, if applicable	Pages 3,4 and top of 5; Table 1; page 5 first paragraph of section on census comparison
Data sources/ measurement	8*	For each variable of interest, give sources of data and details of methods of assessment (measurement). Describe comparability of assessment methods if there is more than one group	Page 2-3; first two paragraphs of sub-section on small area estimation Page 5 first paragraph of section on Census comparison
Bias	9	Describe any efforts to address potential sources of bias	Page 5: use of MCMC modelling
Study size	10	Explain how the study size was arrived at	Not applicable – secondary data analysis of all available data
Quantitative variables	11	Explain how quantitative variables were handled in the analyses. If applicable, describe which groupings were chosen and why	Pages 3,4 and top of 5; Table 1
Statistical methods	12	(*a*) Describe all statistical methods, including those used to control for confounding	Page 5: use of multilevel small area estimation; Page 6 on census comparison
		(*b*) Describe any methods used to examine subgroups and interactions	Page six last paragraph of section on census comparison notes separate analyses for England, Scotland and

For peer review only - http://bmjopen.bmj.com/site/about/guidelines.xhtml

			Wales
		(*c*) Explain how missing data were addressed	Not relevant
		(*d*) *Cross-sectional study*—If applicable, describe analytical methods taking account of sampling strategy	Page 5: use of multilevel modelling to take account of hierarchical sample design in source surveys
		(*e*) Describe any sensitivity analyses	Page 6: this is the purpose of the census comparison
**Results**			
Participants	13*	(a) Report numbers of individuals at each stage of study—eg numbers potentially eligible, examined for eligibility, confirmed eligible, included in the study, completing follow-up, and analysed	Table 1
		(b) Give reasons for non-participation at each stage	Not relevant
		(c) Consider use of a flow diagram	Not relevant
Descriptive data	14*	(a) Give characteristics of study participants (eg demographic, clinical, social) and information on exposures and potential confounders	Table 2
		(b) Indicate number of participants with missing data for each variable of interest	Not relevant
Outcome data	15*	*Cross-sectional study*—Report numbers of outcome events or summary measures	Table 3
		(*a*) Give unadjusted estimates and, if applicable, confounder-adjusted estimates and their precision (eg, 95% confidence interval). Make clear which confounders were adjusted for and why they were included	Table 3
		(*b*) Report category boundaries when continuous variables were categorized	Not relevant
Main results	16	(*c*) If relevant, consider translating estimates of relative risk into absolute risk for a meaningful time period	Not relevant
		Report other analyses done—eg analyses of subgroups and interactions, and sensitivity analyses	Page 8-11, Tables 4 and 5, Figure 1
Other analyses	17	Summarise key results with reference to study objectives	Pages 11-12
**Discussion**			
Key results	18	Give a cautious overall interpretation of results considering objectives, limitations, multiplicity of analyses, results from similar studies, and other relevant evidence	Pages 11
Limitations	19	Discuss the generalisability (external validity) of the study results	Pages 12-13
Interpretation	20	Give a cautious overall interpretation of results considering objectives, limitations, multiplicity of analyses, results from similar studies, and other relevant evidence	Page 13
Generalisability	21	Give the source of funding and the role of the funders for the present study and, if applicable, for the original study on which the present article is based	Page 14
Funding	22		Page 14

For peer review only

BMJ Open

Integrating national surveys to estimate small area variations in poor health and limiting long term illness in Great Britain

Journal:	*BMJ Open*
Manuscript ID	bmjopen-2017-016936.R1
Article Type:	Research
Date Submitted by the Author:	12-May-2017
Complete List of Authors:	Moon, Graham; University of Southampton, Geography and Environment<br>Aitken, Grant; ISD Scotland<br>Taylor, Joanna; Independent Scholar<br>Twigg, Liz; University of Portsmouth
**Primary Subject Heading**:	Research methods
Secondary Subject Heading:	Public health
Keywords:	Small Area Estimation, Survey Methodology, Self-rated General Health, Limiting Long-term Illness

SCHOLARONE™ Manuscripts

For peer review only

Integrating national surveys to estimate small area variations in poor health and limiting long term illness in Great Britain

Graham Moon (*)
Geography and Environment, University of Southampton, Highfield, Southampton SO17 1BJ, UK
(g.moon@soton.ac.uk; tel. 02392 843822)

Grant Aitken
Information Services Division, NHS Scotland, UK

Joanna Taylor
Independent Scholar, Petersfield, UK

Liz Twigg
Department of Geography, University of Portsmouth, UK

(*) Corresponding Author

Keywords

Small Area Estimation; Survey Methodology; Self-rated General Health ; Limiting Long-term Illness

Word count: 3405 excluding title page, references, figures and tables

For peer review only

Integrating national surveys to estimate small area variations in poor health and limiting long term illness in Great Britain

Abstract

Objectives: This study aims to address, for the first time, the challenges of constructing small area estimates of health status using linked national surveys. The study also seeks to assess the concordance of these small area estimates with data from national censuses.
Setting: Population level health status in England, Scotland and Wales
Participants: A linked integrated dataset of 23,374 survey respondents (16+ years) from the 2011 waves of the Health Survey for England (n=8,603), the Scottish Health Survey (n=7,537) and the Welsh Health Survey (n=7,234)
Primary and secondary outcome measures: Population prevalence of poorer self-rated health and limiting long term illness. A multilevel small area estimation modelling approach was used to estimate prevalence of these outcomes for Middle Super Output Areas in England and Wales and Intermediate Zones in Scotland. The estimates were then compared to matched measures from the contemporaneous 2011 UK Census.
Results: There was a strong positive association between the small area estimates and matched Census measures for all three countries for both poorer self-rated health (r=0.828, 95% CI 0.821 - 0.834) and limiting long-term illness (r=0.831, 95% CI 0.824 to 0.837), although systematic differences were evident and small area estimation tended to indicate higher prevalences than Census data.
Conclusions: Despite strong concordance, variations in the small area prevalences of poorer self-rated health and limiting long-term illness evident in Census data cannot be replicated perfectly using small area estimation with linked national surveys. This reflects a lack of harmonisation between surveys over question wording and design. The nature of small area estimates as 'expected values' also needs to be better understood.

Article Summary

Strengths and limitations of this study

- Reports on small area estimates of health status derived from the novel linkage of three large well-designed routine national health surveys
- Develops and enhances a well-reputed approach to the small area estimation of health indicators
- Highlights the enduring challenges entailed in harmonising measures of health status across different surveys
- Enhances understanding of the reasons that underlie discrepancies between small area estimates and equivalent observed data

For peer review only

Introduction

Small area data on variations in health status have long been seen as central to geographical comparisons of health needs [1]. Effectiveness is enhanced when data are available at very local scales facilitating sensitive, community-focussed action. Such data additionally need to be provided to consistent, robust and reliable standards that are shared nationally and, ideally, internationally. Moreover they need to capture the health status of whole populations, to ensure comprehensive rather than selective coverage. These ideals are seldom met. National surveys are not usually designed to provide valid data down to suitably small areas, although an exception is offered in the few cases where national censuses provide small area health data. Local surveys, if they exist at all, can vary substantially in design, making comparisons difficult. Using administrative data to make local estimates restricts attention to users of services and thus lacks population representativeness.

To address the unmet need for small area data, researchers across the world have increasingly turned to small area estimation (SAE) methods [2]. SAE methods use statistical or mathematical methods to manipulate national survey data to produce local estimates for a target measure. A common methodological root is the association in a national survey between a target variable of interest and covariates thought to predict that target variable. Estimation uses local data on the covariates. The SAE process generally takes place within a single national setting with multiple surveys being used to enhance the pool of available covariates. There has been limited attention to linking surveys from different geographical contexts to enhance the spatial coverage of estimates through a larger pool of cases. Addressing this omission recognises that health issues seldom respect geographical borders. It also enables the identification of 'place effects' on outcome measures and reveals the extent to which definitions of key health variables differ with geographical setting.

Health applications of SAE have focussed mainly on outcomes and behaviours [3-7]. Rather less attention has been given to SAEs of general measures of health status such as Limiting Long-Term Illness (LLTI) and Self-reported General Health (SRGH). Such measures are good at picking up ageing

For peer review only - http://bmjopen.bmj.com/site/about/guidelines.xhtml

populations and chronic illness as well as pockets of severe health deprivation [8-13]. For these reasons, they are frequently included in national censuses. Though this might be held to obviate the need for SAEs of health status, it presents an opportunity to address a frequent criticism of SAEs: the absence of validation. Small area census data on health status provide what is arguably a 'gold standard' against which SAEs can be compared [14].

Motivated by the lack of previous research on health-related SAE using surveys from more than one geographical setting and the opportunity to ground-truth SAEs of health status using small area census data, this paper addresses two objectives. First, we develop parsimonious SAE models of LLTI and SRGH using surveys from multiple geographical settings. Second, the estimates from these models are compared to population census data. As both SAEs and census data are population-level measures, we hypothesise a close concordance.

Methods

We created SAEs by modelling routine data and applying the coefficients from our models to census data on the covariate measures. Our SAEs were then compared to census data on LLTI and SRGH. Great Britain was the setting for the study as we had ready access to the separate surveys for the constituent countries of Wales, Scotland and England as well as to census data for each country. LLTI and SRGH are established measures of health status in each country. The UK Census began collecting information on SRGH and LLTI in 1991.

Small Area Estimation

SAE models were derived primarily from a linked data file comprising the individual responses to the 2011 versions of the Health Survey for England (HfSE), the Scottish Health Survey (SHS) and the

For peer review only - http://bmjopen.bmj.com/site/about/guidelines.xhtml

Welsh Health Survey (WHS). These sources were accessed through the UK Data Service. Surveys from 2011 were selected to facilitate direct comparison with 2011 decennial population census. Full details of the conduct of the surveys, the measurement of variables and response rates are given in the 2011 HSfE, SHS and WHS reports [15-17]. Collectively the sources offer comprehensive well-found authoritative insights into health status in each country. After data cleaning to address missing data the working data file comprised 23,374 individuals (aged 16+ years; England n=8,603, Scotland n=7,537, Wales n=7,234).

Data from the three health surveys were supplemented with linked data on disability and multiple deprivation at the area level, in recognition of the close association between these factors and our target variable [8, 10, 18]. We obtained special permission to use geo-coded data to link these additional variables for Middle (layer) Super Output Areas (MSOAs). MSOAs (known as Intermediate Zones in Scotland, but referred to as MSOAs throughout this paper) total 6,791 in England, 1,235 in Scotland and 410 in Wales. They are small areas ranging in population from 5,000-15,000 in England and Wales, and 2,500-6,000 in Scotland. We added the combined rate of Disability Living Allowance and Attendance Allowance per 1,000 adults per MSOA, and the combined rate of Incapacity Benefit plus Severe Disablement Allowance plus Employment and Support Allowance per 1,000 adults per MSOA. Both benefit measures were derived from data held on the NOMIS website [19]. For deprivation we used a UK-wide Index of Multiple Deprivation (IMD) score, calibrated from the English, Scottish and Welsh versions of the index using a modified version of the method outlined by Payne and Abel. [20]. To track survey effects we also added a flag denoting whether a respondent was from England, Scotland or Wales.

The outcome measures for our two SAE models were poorer SRGH and possession of an LLTI. Both measures required harmonisation of the relevant questions across the three surveys and in the Decennial Population Census (Table 1). For SRGH, respondents were asked to self-report their general health. In the Census, HSfE and SHS 'very good' or 'good' general health was coded as good

For peer review only - http://bmjopen.bmj.com/site/about/guidelines.xhtml

SRGH; ‘fair’, ‘bad’ or ‘very bad’ general health was coded as poorer SRGH. In the WHS ‘excellent’, ‘very good’ or ‘good’ general health was coded as good SRGH and ‘fair’ or ‘poor’ general health was coded as poorer SRGH. LLTI was also dichotomised. In the Census and the WHS, respondents were asked first to define if they had any long-term illness, health problem or disability which limited their daily activities or the work they could do. If they responded ‘yes, limited a lot’ or ‘yes, limited a little’ they were coded as having a LLTI, and if they responded ‘no’ they were not. In the HSfE people were asked if they had a long-standing illness, disability or infirmity troubling them over a period of time. If they responded yes, they were then asked if this limited their activities in any way, with those individuals answering yes to this second question being coded as having a LLTI. In the SHS people were if they had a long-standing physical or mental condition or disability that troubled them for at least 12 months. If they responded yes, they were asked if this condition limited their activities in any way, with those individuals answering yes to this question coded as having a LLTI.

Table 1: Wording of SRGH and LLTI questions for Census, HSfE, SHS and WHS for people aged 16+

	**SRGH: question and responses**			
**2011 Census**	How is your health in general?	Very Good or Good	Fair	Bad or Very Bad
**2011 Health Survey for England**	How is your health in general? Would you say it was….	Very Good or Good	Fair	Bad or Very Bad
**2011 Scottish Health Survey**	How is your health in general? Would you say it was…..	Very Good or Good	Fair	Bad or Very Bad
**2011 Welsh Health Survey**	In general, would you say your health is?	Excellent, Very Good or Good	Fair	Poor
	**LLTI: question and responses**			
**2011 Census**	Are your day-to-day activities limited because of a health problem or disability which has lasted, or is expected to last, at least 12 months? Include problems related to old age.	No	Yes, limited a little	Yes, limited a lot
**2011 Health Survey for England**	Do you have any long-standing illness, disability or infirmity? By long-standing I mean anything that has troubled you over a period of time, or that is likely to affect you over a period of time?	No	Yes	
	If yes, does this illness or disability/do any of these illnesses or disabilities limit your activities in any way?	No	Yes	
**2011 Scottish Health Survey**	Do you have a long-standing physical or mental condition or disability that has troubled you for at least 12 months, or that is likely to affect you for at least 12 months?	No	Yes	
	If yes, does (name of condition) limit your activities in any way?	No	Yes	
**2011 Welsh Health Survey**	Are your day-to-day activities limited because of a health problem or disability which has lasted, or is expected to last, at least 12 months? Include problems related to old age.	No	Yes, limited a little	Yes, limited a lot

For peer review only - http://bmjopen.bmj.com/site/about/guidelines.xhtml

Covariate data to facilitate the generation of small area estimates were derived from the linked data file. A parsimonious selection of covariates was made reflecting the known associations between LLTI and SRGH and age, sex, disability and deprivation [21, 22]. Individual-level age and sex were self-reported measures. Age was grouped into thirteen categories: those aged 16 to 19; 5 year age groups until the age of 74; and all individuals aged 75+. These individual-level covariates were supplemented with the area-level measures of disability and deprivation introduced above.

To create SAEs we used the established multilevel small area estimation process recognising the hierarchical structure of the source surveys [3, 23, 24]. This approach involves developing a multilevel model using survey data with covariate terms that are also available for all target small areas. The process began with initial data management into individual and area level covariates using IBM SPSS Statistics (V22). Area-level variables were centred on their grand mean. We then developed 2-level logistic models of individuals nested within MSOAs using MLwiN (V2.35) [25, 26]. Separate models were produced to estimate SRGH and LLTI. Models were tested for interactions between the age and sex terms, and variables were retained in the final models if they were found to be statistically significant using the Chi-Squared test ($p \leq 0.05$). We considered modelling with the individual non-response weights available in each of the national surveys but, after exploration, elected to proceed with unweighted data. Views vary on whether or not to use weighted data in small area estimation with the Bayesian nature of our modelling process offering support for our decision [27].

Our SAE models were initially estimated using iterative generalised least squares (IGLS) with first order maximum quasi-likelihood estimation and took the general form:

$$Y_{ij} = \beta_{0j} + \boldsymbol{\beta_{1ij}} + \boldsymbol{\beta_{2ij}} + \mu_{0j}$$

where Y represents the outcome, whether an individual has an LLTI or is in poor health, β_{0j} is the intercept in the model, $\boldsymbol{\beta_{1ij}}$ represents covariates measured at the individual level (age and sex), $\boldsymbol{\beta_{2j}}$ refers to covariates measured at the MSOA level (benefit receipts, IMD and the flag denoting

For peer review only - http://bmjopen.bmj.com/site/about/guidelines.xhtml

England, Scotland or Wales), and μ_{0j} indicates the MSOA level variance; individual level variance is constrained to one in the binomial model. Once the IGLS models achieved convergence, their coefficients were used as informative priors in Bayesian Monte Carlo Markov Chain (MCMC) models to allow for more robust estimates and standard errors. Both MCMC models were run through 500,000 iterations, with an initial burn-in period of 50,000 iterations. The SAE process concluded with the generation of SAEs of SRGH and LLTI at MSOA level produced by converting the final MCMC model logit coefficients to probabilities and applying them to a data file for all MSOAs in Great Britain comprising cross-tabulations of age and sex from the 2011 Decennial Population Census together with the area-level indicators.

Census Comparison

In the UK, the 2011 Census is the most recent source of information on local inequalities in SRGH and LLTI. We compared our MSOA SAEs to census data on SRGH and LLTI for MSOAs. For England and Wales this was sourced from the ONS official labour market statistics website. For Scotland it was downloaded from the Scottish Census data warehouse.

SAEs and Census estimates were compared using regression and correlation analysis following the Scarborough methodology used previously for validating SAEs for coronary heart disease [28]. SAEs were plotted against the Census measurement of the same target variable at the MSOA level. Convergent validity was achieved if the line of best fit had a gradient with confidence intervals including one and an intercept with confidence intervals including zero. For each model, we considered four regression lines: one for Great Britain as a whole and one each for the constituent countries of England, Scotland and Wales. We also sought high correlations.

For peer review only - http://bmjopen.bmj.com/site/about/guidelines.xhtml

Results

A descriptive summary of the full linked data file used to construct the SAEs is set out in Table 2. The resulting small area estimation models are shown in Table 3. We show logits and standard errors as these provide the input to the SAE process. Having poor SRGH was largely a function of increasing age, and higher MSOA IMD scores (increased deprivation). A similar picture was evident for LLTI with being female playing an additional role. MSOA-level benefit measures had little effect on either SRGH or LLTI. For SRGH the models showed no difference between Scotland and the reference country of England but a markedly lower likelihood of poorer SRGH in Wales. In contrast, being located in either Wales or Scotland was associated with a higher likelihood of reporting an LLTI compared to England. Both models were relatively successful in capturing variation in their outcome measures, with the LLTI model being marginally more effective.

For peer review only

For peer review only - http://bmjopen.bmj.com/site/about/guidelines.xhtml

Table 2: Descriptive statistics: full linked data set

Variable	Category	Full Sample		General Health		LLTI	
		Number	Column %	Number	Column %	Number	Column %
**All**	**Aged 16+**	23 374	100	23 355	100	23 281	100
**Country**	**England**	8 603	36.8	8 603	36.8	8 603	37.0
	**Scotland**	7 537	32.3	7 537	32.3	7 537	32.4
	**Wales**	7 234	30.9	7 215	30.9	7 141	30.5
**Sex**	**Male**	10 285	44.0	10 276	44.0	10 240	44.0
	**Female**	13 089	56.0	13 079	56.0	13 041	56.0
**Age**	**16-19**	772	3.3	770	3.3	780	3.4
	**20-24**	1 146	4.9	1 146	4.9	1 153	5.0
	**25-29**	1 403	6.0	1 403	6.0	1 404	6.0
	**30-34**	1 650	7.1	1 650	7.1	1 645	7.1
	**35-39**	1 790	7.7	1 788	7.7	1 783	7.7
	**40-44**	2 123	9.1	2 122	9.1	2 112	9.1
	**45-49**	2 140	9.2	2 138	9.2	2 136	9.2
	**50-54**	2 032	8.7	2 030	8.7	2 023	8.7
	**55-59**	1 941	8.3	1 940	8.3	1 935	8.3
	**60-64**	2 272	9.7	2 271	9.7	2 268	9.7
	**65-69**	1 917	8.2	1 914	8.2	1 907	8.2
	**70-74**	1 509	6.5	1 508	6.5	1 494	6.4
	**75+**	2 679	11.4	2 675	11.4	2 641	11.3
**General Health**	**Fair/poor/ very poor**	-	-	5 956	25.5	-	-
	**Very good/ good**	-	-	17 399	74.5	-	-
**LLTI**	**Present**	-	-	-	-	7 309	31.4
	**Not present**	-	-	-	-	15 972	68.6
		**Mean (SD)**		**Mean (SD)**		**Mean (SD)**	
**Disability Benefits**		109.39 (±45.19)		109.38 (±45.20)		109.18 (±45.13)	
**Work Benefits**		86.66 (±57.49)		86.68 (±57.51)		86.73 (±57.60)	
**IMD**		19.11 (±10.67)		19.10 (±10.67)		19.07 (±10.67)	

For peer review only

For peer review only - http://bmjopen.bmj.com/site/about/guidelines.xhtml

Table 3: SRGH and LLTI model parameters: logit and standard error values

	Poorer SRGH model		LLTI model	
	Logit	Standard Error	Logit	Standard Error
**Constant**	-2.470	0.137	-2.422	0.122
**Sex: Female**	0.059	0.032	0.151	0.031
**Age: 20-24**	0.188	0.165	-0.193	0.156
**Age: 25.29**	0.339	0.157	0.178	0.143
**Age: 30-34**	0.208	0.156	0.173	0.140
**Age: 35-39**	0.680	0.148	0.455	0.135
**Age: 40-44**	0.863	0.144	0.654	0.131
**Age: 45-49**	1.168	0.142	0.984	0.129
**Age: 50-54**	1.465	0.142	1.281	0.128
**Age: 55-59**	1.650	0.142	1.436	0.128
**Age: 60-64**	1.758	0.140	1.671	0.127
**Age: 65-69**	1.886	0.141	1.816	0.128
**Age: 70-74**	2.086	0.143	2.023	0.130
**Age: 75+**	2.541	0.138	2.617	0.126
**IMD**	0.023	0.003	0.011	0.003
**Disability Benefits**	0.002	0.001	0.002	0.001
**Work Benefits**	-0.001	0.001	-	-
**Scotland**	0.150	0.110	0.319	0.046
**Wales**	-0.456	0.056	0.469	0.049
**Deviance Information Criterion Reduction (1)**	2179		3031	
**Explained Variance (2)**	15.6%		19.5%	

Notes:
Greyed out data are not statistically significant (p>0.05; Wald test). The models' constants are a man aged 16-19, living in an MSOA of average IMD, disability receipt and work benefit receipt, and resident in England.

(1) Deviance Information Criterion reductions from null models. Larger reductions indicate better models [29]
(2) Computed following Snijders and Bosker latent variable approach [30].

Summary statistics on the SAEs derived from these models are presented for England, Scotland and Wales in Table 4. The mean MSOA prevalence of poorer SRGH is highest in Scotland and lowest in Wales. For LLTI, Wales has the highest mean MSOA prevalence and England is lowest. In both Scotland and Wales, the mean MSOA prevalence for LLTI is notably higher than that for poorer SRGH. Table 4 also indicates the mean MSOA census prevalences for poorer SRGH and LLTI for each country, providing initial insights into the match between SAEs and 'gold standard' census data. Differences are evident. For poorer SRGH, Scotland has the lowest mean prevalence on the Census measure but

For peer review only - http://bmjopen.bmj.com/site/about/guidelines.xhtml

the highest on SAE measure; for Wales the situation is reversed. Both England and Scotland have a lower mean prevalence on the Census measure compared to the SAE while for Wales the SAE prevalence is higher. In the case of LLTI the relative position of the three countries is the same for the two measures but the Census mean prevalences are substantially lower.

Table 4: MSOA level SAEs and Census estimates compared

	Poorer SRGH					LLTI				
	Census mean	SAE				Census mean	SAE			
		Mean	SD	Min	Max		Mean	SD	Min	Max
**England**	22.04	25.92	5.53	10.64	55.76	21.48	25.23	4.09	9.4	43.96
**Scotland**	21.33	29.17	6.59	11.89	54.62	23.10	31.47	4.86	14.31	48.33
**Wales**	26.23	21.65	5.23	6.68	41.36	27.76	37.29	4.97	13.97	51.54

All figures percentages except SD (standard deviation)

The concordance between SAEs and Census data is explored further in Figure 1. The diagonal reference line captures the scenario where the SAE and Census estimates would match. The SAEs for poorer SRGH in England and Scotland were higher for most small areas compared to the Census, while the great majority of Welsh estimates were lower. For LLTI most SAE prevalences exceeded the equivalent Census prevalences; those MSOAs with SAEs less than the corresponding Census values tended to be in England.

Figure 1: SAEs versus Census Estimates: LLTI (a), poorer SRGH (b)

insert bmjopenfigure1a.tif
Insert bmjopenfigure1b.tif>>

For peer review only

Table 5 continues the exploration of the match between SAEs and Census using the 'Scarborough criteria'. There are strong correlations between the two measures across all settings suggesting broad agreement. However, neither for individual countries nor collectively do any of the SAEs exhibit anything approaching the requirements for a strong concordance with Census measures. All have intercepts significantly above zero and gradients that depart markedly from one.

Table 5: Convergent validity of SAEs against Census estimates at MSOA level

	Intercept	Lower CI	Upper CI	Contain Zero?	Gradient	Lower CI	Upper CI	Contain One?	Correlation
**Poorer SRGH**									
**GB**	6.87	6.58	7.16	X	0.87	0.86	0.89	X	0.828**
**England**	4.98	4.75	5.21	X	0.95	0.94	0.96	X	0.916**
**Scotland**	8.64	8.18	9.10	X	0.96	0.94	0.98	X	0.933**
**Wales**	3.76	2.04	5.49	X	0.68	0.62	0.75	X	0.719*
**LLTI**									
**GB**	9.24	8.99	9.50	X	0.79	0.78	0.81	X	0.832**
**England**	10.56	10.36	10.74	X	0.68	0.67	0.69	X	0.895**
**Scotland**	13.31	12.87	13.74	X	0.79	0.77	0.81	X	0.922**

For peer review only - http://bmjopen.bmj.com/site/about/guidelines.xhtml

**Wales**	17.98	16.48	19.47	X	0.69	0.64	0.75	X	0.789*

*significant p<0.05
**significant p<0.01

Discussion

Even with jurisdictions as closely associated as England, Scotland and Wales and ongoing UK-wide attempts at harmonising questions in health and other routine national surveys, there remain differences in wording and format that pose difficulties for SAE when it comes to linking survey input data across geographical settings. Overcoming these difficulties requires compromise and accommodation but is possible. In response to our first objective, we have shown how a cross-setting dataset can be developed as the basis for a parsimonious SAE model reflecting the key social determinants of poorer SRGH and LLTI.

Our second objective entailed comparing our SAEs with matched and contemporaneous MSOA Census data. Here our results were at best equivocal. While strong correlations were evident, suggesting a tight association between the two measures running in an expected direction, closer inspection revealed that SAEs tended broadly to exceed Census estimates, and MSOAs in different national contexts returned starkly varying results across the two measures. It would, of course, be possible, in the case of this specific example, to go further and weight the SAEs with the Census data to bring the SAEs closer to the presumed (Census) gold standard. More generally however such post hoc adjustment presumes the availability of gold standard data and, in most cases, SAEs are created to fill the gaps occasioned by the absence of such data at a local geographical scale making. While post hoc adjustment at a local level is therefore seldom possible, it can be done when gold standard data are available at a higher spatial level, ensuring for example that local SAEs sum to known higher level figures.

Despite this lack of concordance, our findings update previous knowledge on SRGH and LLTI in Great Britain. Levels of LLTI were found to be higher in Wales than in Scotland or England using 1991

For peer review only - http://bmjopen.bmj.com/site/about/guidelines.xhtml

Census data [31]. This situation still persists and is evident in our SAEs, which run counter to suggestions that models under-predict LLTI in Wales but possibly confirm a continued tendency to over-prediction in Scotland [32]. 2001 Census data was used to highlight higher rates of poor SRGH in Wales compared to the rest of Great Britain [33]. Another study with 2001 Census data confirmed that levels of poorer SRGH were worst in Wales, followed by Scotland, then England [34]. Our analysis sustains this finding with 2011 Census data but our SAEs suggest a contrary picture in which Wales has better levels of poorer SRGH than either Scotland or England.

Reasons for the differences between our SAEs and contemporaneous Census data for matched measures are of relevance to future research on SAE and merit examination. Differences in the wording of the SRGH and LLTI questions were evident between the source surveys and between the surveys and the Census. There were also variations in the categorisation of outcome possibilities and in the positioning of the questions within the surveys. In the English and Scottish surveys the SRGH question appeared at the start of the individual interview immediately after age, with the LLTI question appearing immediately afterwards . In the WHS, additional health questions were asked before the SRGH questions . Asking about SHRG after, rather than before, other health questions, results in more positive health assessments and replacing 'very good' with 'excellent' as a response category has a similarly more positive effect [35]. The wording of the WHS question about LLTI and specifically the inclusion of the reference to 'problems of old age' may have led to greater levels of problem identification [9] [11]. Collectively there points echo calls from other jurisdictions for continued work on the harmonisation of questions between surveys and censuses across national borders [36].

Another reason for discrepancies between our SAEs and Census data is that Census and survey information are collected in different ways. The householder is responsible for ensuring that the UK Census is completed whereas the surveys are completed by an individual. Furthermore the UK Census is a self-completion form but our surveys were interviewer administered. Both these factors

For peer review only - http://bmjopen.bmj.com/site/about/guidelines.xhtml

can lead to Census prevalence data generally being lower that that from surveys [37]. One manifestation of both this and the previous point is that the input survey data for our SAE models (Table 2) collectively evidenced prevalences of 25.5% for poorer SRGH and 31.4% for LLTI, both greater than all bar one of the corresponding national prevalences evident for Census data in Table 3. Inevitably the SAEs accord more with this input data.

Though soundly based on theory, our models were undeniably simple. Research using a multinomial LLTI outcome measures has found improved concordance with 2011 Census estimates at MSOA level in England [38]. Additional socio-economic, health and clinical covariates, known to be associated with increased SRGH or LLTI prevalence were not included within the modelling framework due to data constraints and the requirements of the SAE process. These omissions may have been significant and uncaptured by the areal IMD and benefit measures. For example there may be systematic differences between social groups in their understanding of SRGH and the omission of ethnicity is undoubtedly significant [39].

As a final point, we reflect on the nature of SAEs and Census data. SAEs are perhaps most usefully seen as expected values for the target variable, given the covariates included in the modelling process [40, 41]. Census data are also estimates, subject to differential response and variations in understanding [42, 43]. The Census is thus not a 'gold standard' though it comes close. We should not anticipate that SAEs should have to match exactly to the estimates provided by their equivalent census measures. Rather, SAEs provide an expectation against which Census data can be compared. In our analysis, linking surveys across three countries thus points to an expectation that levels of poorer SRGH and LLTI should be rather higher than suggested by Census data, given variations in population distributions by age and sex, and the varying areal prevalences of benefit take-up and deprivation. .

For peer review only - http://bmjopen.bmj.com/site/about/guidelines.xhtml

Funding Statement

This work was supported by the UK Economic and Social Research Council grant number ES/K003046/1.

Author Contributions

GM and LT conceived and led the study, and participated in data analysis and interpretation. JT led on data acquisition and participated in data analysis. GA participated in data analysis and interpretation. All authors contributed to drafting, revision and final approval of the manuscript. All authors are responsible for the manuscript.

Competing Interests

There were no competing interests

Data Sharing

Project data files are available by emailing the corresponding author

For peer review only

References

1 Mooney A, Rives N. Measures of community health status for health planning. *Health Services Research* 1978;**13**:129-45.

2 Rao J, Molina I. *Small Area Estimation*. London: John Wiley & Sons 2015.

3 Twigg L, Moon G. Predicting small area health-related behaviour: a comparison of multilevel synthetic estimation and local survey data. *Social Science and Medicine* 2002;**54**:931-7.

4 Dwyer-Lindgren L, Mokdad AH, Srebotnjak T*, et al.* Cigarette smoking prevalence in US counties: 1996-2012. *Population Health Metrics* 2014;**12**.

5 Ha NS, Lahiri P, Parsons V. Methods and results for small area estimation using smoking data from the 2008 National Health Interview Survey. *Statistics in Medicine* 2014;**33**:3932-45.

6 Wang Y, Ponce NA, Wang P*, et al.* Generating health estimates by zip code: A semiparametric small area estimation approach using the California health interview survey. *American Journal of Public Health* 2015;**105**:2534-40.

7 Zhang X, Holt JB, Yun S*, et al.* Validation of Multilevel Regression and Poststratification Methodology for Small Area Estimation of Health Indicators from the Behavioral Risk Factor Surveillance System. *American Journal of Epidemiology* 2015;**182**:127-37.

8 Marshall A. Developing a methodology for the local estimation and projection of limiting long term illness and disability. *PhD Thesis*: University of Manchester 2009.

9 Cohen G, Forbes J, Garraway M. Interpreting self reported limiting long term illness. *BMJ* 1995;**311**:722.

10 Martin S, Sheldon TA, Smith P. Interpreting the new illness question in the UK census for health research on small areas. *Journal of Epidemiology and Community Health* 1995;**49**:634-41.

11 Jordan K, Ong BN, Croft P. Researching limiting long-term illness. *Social Science and Medicine* 2000;**50**:397-405.

12 Jordan K, Ong BN, Croft P. Previous consultation and self reported health status as predictors of future demand for primary care. *Journal of Epidemiology and Community Health* 2003;**57**:109-13.

13 Taylor J, Twigg L, Moon G. The convergent validity of three surveys as alternative sources of health information to the 2011 UK census. *Social Science and Medicine* 2014;**116**:187-92.

14 Taylor J, Moon G, Twigg L. Using geocoded survey data to improve the accuracy of multilevel small area synthetic estimates. *Social Science Research* 2016;**56**:108-16.

15 NatCen Social Research. Health Survey for England 2011 user guide. London: NatCen 2012.

16 Rutherford L, Sharp C, Bromley C, eds. *The Scottish Health Survey 2011 edition volume 3 technical report*. Edinburgh: A National Statistics Publication for Scotland 2012.

17 NatCen Social Research. Welsh Health Survey 2012 - Technical Report London: NatCen 2012a.

18 Payne N, Saul C. What common disorders do those reporting limiting long-term illness experience, and what is their survival and health service utilization experience? *Journal of Public Health Medicine* 2000;**22**:324-9.

19 NOMIS. Benefit Claimants: working age clients for small areas. https://www.nomisweb.co.uk/query/construct/summary.asp?mode=construct&version=0&dataset=103.

20 Payne R, Abel G. UK indices of multiple deprivation - a way to make comparisons across constituent countries easier. London: Office for National Statistics 2012.

21 Martin D, Wright JA. Disease prevalence in the English population: A comparison of primary care registers and prevalence models. *Social Science and Medicine* 2009;**68**:266-74.

22 Zhang X, Cook P, Lisboa P*, et al.* The effects of deprivation and relative deprivation on self-reported morbidity in England: An area-level ecological study. *International Journal of Health Geographics* 2013;**12**:5.

For peer review only - http://bmjopen.bmj.com/site/about/guidelines.xhtml

23 Twigg L, Moon G, Jones K. Predicting small-area health-related behaviour: a comparison of smoking and drinking indicators. *Social Science & Medicine* 2000;**50**:1109-20.

24 Moon G, Quarendon G, Barnard S*, et al.* Fat nation: Deciphering the distinctive geographies of obesity in England. *Social Science and Medicine* 2007;**65**:20-31.

25 Rasbash J, Charlton C, Browne W*, et al.* MLwiN Version 2.35. Centre for Multilevel Modelling, University of Bristol. 2015.

26 Browne W. MCMC Estimation in MLwiN. Centre for Multilevel Modelling, University of Bristol 2009.

27 Gelman A. Struggles with survey weighting and regression modeling. *Statistical Science* 2007;**22**:153-64.

28 Scarborough P, Allender S, Rayner M*, et al.* Validation of model-based estimates (synthetic estimates) of the prevalence of risk factors for coronary heart disease for wards in England. *Health & Place* 2009;**15**:596-605.

29 Browne W. *MCMC Estimation Methods in MLwiN*. University of Bristol: Centre for Multilevel Modelling 2015.

30 Snijders T, Bosker R. Multilevel analysis: An introduction to basic and applied multilevel analysis. London: Sage 1999.

31 Gould M, Jones K. Analyzing perceived limiting long-term illness using UK census microdata. *Social Science & Medicine* 1996;**42**:857-69.

32 Senior ML. Area Variations in Self-perceived Limiting Long Term Illness in Britain, 1991: Is the Welsh Experience Exceptional?, . *Regional Studies* 1998;**32**:265-80.

33 Doran T. Is there a north-south divide in social class inequalities in health in Great Britain? Cross sectional study using data from the 2001 census. *BMJ* 2004:1043.

34 O'Reilly D. Self reported health and mortality: ecological analysis based on electoral wards across the United Kingdom. *BMJ* 2005;**331**:938.

35 Bowling A, Windsor J. The effects of question order and response-choice on self-rated health status in the English Longitudinal Study of Ageing (ELSA). *J Epidemiol Community Health* 2008;**62**:81-5.

36 Desesquelles AF, Egidi V, Salvatore MA. Why do Italian people rate their health worse than French people do? An exploration of cross-country differentials of self-rated health. *Social Science & Medicine* 2009;**68**:1124-8.

37 Gnambs T, Kaspar K. Disclosure of sensitive behaviors across self-administered survey modes: A meta-analysis. *Behavior Research Methods* 2015;**47**:1237-59.

38 Moon G, Twigg L, Taylor J. Estimating local variations in the prevalence of limiting long-term illness: evaluating multinomial small area synthetic estimation. *Journal of Epidemiology and Community Health* 2014;**68**:A71-A.

39 Evandrou M, Falkingham J, Feng Z*, et al.* Ethnic inequalities in limiting health and self-reported health in later life revisited. *Journal of Epidemiology and Community Health* 2016;**70**:653-62.

40 Twigg L, Moon G, Walker S. The smoking epidemic in England. London: Health Development Agency 2004.

41 Moon G, Altankhuyag G, Barnard S*, et al.* Smoking Prevalence in Scotland: 2003/4 sub-national estimates. NHS Health Scotland, Glasgow 2006.

42 Baffour B, Valente P. An evaluation of census quality. *Statistical Journal of the IAOS* 2012;**28**:121-35.

43 Coleman D. The twilight of the census. *Population and Development Review* 2013;**38**:334-51.

For peer review only - http://bmjopen.bmj.com/site/about/guidelines.xhtml

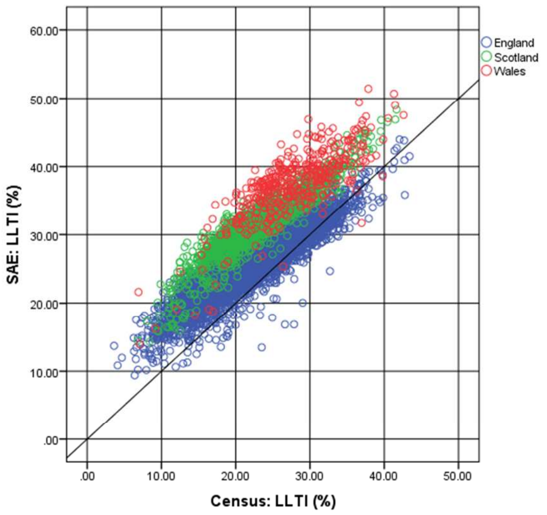

Figure 1: SAEs versus Census Estimates: LLTI (a), poorer SRGH (b)

254x190mm (300 x 300 DPI)

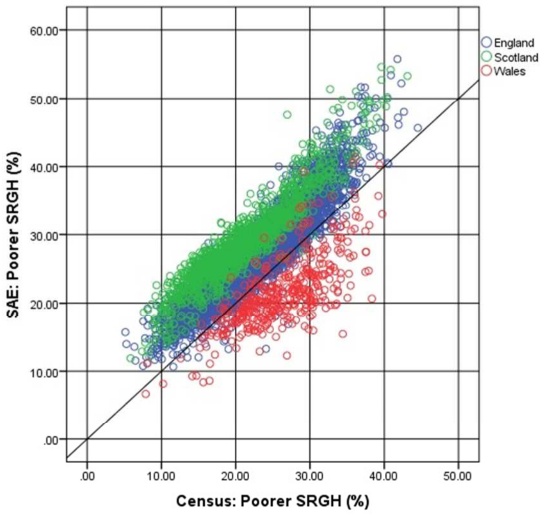

254x190mm (300 x 300 DPI)

STROBE Statement—checklist of items that should be included in reports of observational studies

All items checked and present – subject to adjustment for small area estimation studies

	Item No	Recommendation	Where evident
**Title and abstract**	1	(*a*) Indicate the study's design with a commonly used term in the title or the abstract	Title conveys focus on survey linkage and small area estimation
		(*b*) Provide in the abstract an informative and balanced summary of what was done and what was found	Abstract follows required format
**Introduction**			
Background/rationale	2	Explain the scientific background and rationale for the investigation being reported	Pages 1-2
Objectives	3	State specific objectives, including any prespecified hypotheses	Page 2
**Methods**			
Study design	4	Present key elements of study design early in the paper	Page 2 first paragraph of Methods section
Setting	5	Describe the setting, locations, and relevant dates, including periods of recruitment, exposure, follow-up, and data collection	Page 2-3 first two paragraphs of sub-section on small area estimation, page 5/6 on census comparison
Participants	6	*Cross-sectional study*—Give the eligibility criteria, and the sources and methods of selection of participants	Page 2-3 first two paragraphs of sub-section on small area estimation; Page 5/6 on census comparison
Variables	7	Clearly define all outcomes, exposures, predictors, potential confounders, and effect modifiers. Give diagnostic criteria, if applicable	Pages 3,4 and top of 5; Table 1; page 5 first paragraph of section on census comparison
Data sources/ measurement	8*	For each variable of interest, give sources of data and details of methods of assessment (measurement). Describe comparability of assessment methods if there is more than one group	Page 2-3; first two paragraphs of sub-section on small area estimation Page 5 first paragraph of section on Census comparison
Bias	9	Describe any efforts to address potential sources of bias	Page 5: use of MCMC modelling
Study size	10	Explain how the study size was arrived at	Not applicable – secondary data analysis of all available data
Quantitative variables	11	Explain how quantitative variables were handled in the analyses. If applicable, describe which groupings were chosen and why	Pages 3,4 and top of 5; Table 1
Statistical methods	12	(*a*) Describe all statistical methods, including those used to control for confounding	Page 5: use of multilevel small area estimation; Page 6 on census comparison
		(*b*) Describe any methods used to examine subgroups and interactions	Page six last paragraph of section on census comparison notes separate analyses for England, Scotland and

For peer review only - http://bmjopen.bmj.com/site/about/guidelines.xhtml

			Wales
		(*c*) Explain how missing data were addressed	Not relevant
		(*d*) *Cross-sectional study*—If applicable, describe analytical methods taking account of sampling strategy	Page 5: use of multilevel modelling to take account of hierarchical sample design in source surveys
		(*e*) Describe any sensitivity analyses	Page 6: this is the purpose of the census comparison
**Results**			
Participants	13*	(a) Report numbers of individuals at each stage of study—eg numbers potentially eligible, examined for eligibility, confirmed eligible, included in the study, completing follow-up, and analysed	Table 1
		(b) Give reasons for non-participation at each stage	Not relevant
		(c) Consider use of a flow diagram	Not relevant
Descriptive data	14*	(a) Give characteristics of study participants (eg demographic, clinical, social) and information on exposures and potential confounders	Table 2
		(b) Indicate number of participants with missing data for each variable of interest	Not relevant
Outcome data	15*	*Cross-sectional study*—Report numbers of outcome events or summary measures	Table 3
		(*a*) Give unadjusted estimates and, if applicable, confounder-adjusted estimates and their precision (eg, 95% confidence interval). Make clear which confounders were adjusted for and why they were included	Table 3
		(*b*) Report category boundaries when continuous variables were categorized	Not relevant
Main results	16	(*c*) If relevant, consider translating estimates of relative risk into absolute risk for a meaningful time period	Not relevant
		Report other analyses done—eg analyses of subgroups and interactions, and sensitivity analyses	Page 8-11, Tables 4 and 5, Figure 1
Other analyses	17	Summarise key results with reference to study objectives	Pages 11-12
**Discussion**			
Key results	18	Give a cautious overall interpretation of results considering objectives, limitations, multiplicity of analyses, results from similar studies, and other relevant evidence	Pages 11
Limitations	19	Discuss the generalisability (external validity) of the study results	Pages 12-13
Interpretation	20	Give a cautious overall interpretation of results considering objectives, limitations, multiplicity of analyses, results from similar studies, and other relevant evidence	Page 13
Generalisability	21	Give the source of funding and the role of the funders for the present study and, if applicable, for the original study on which the present article is based	Page 14
Funding	22		Page 14

For peer review only
